# Protocol Study: Resistance Training Program, Nutritional, Sleep, and Screen Use Recommendations in Schoolchildren from Educational Centers in the Extreme South of Chile

**DOI:** 10.3390/mps6050074

**Published:** 2023-08-25

**Authors:** Javier Albornoz-Guerrero, Olga Barceló, Sonia García-Merino, Guillermo García-Pérez-de-Sevilla, Igor Cigarroa, Rafael Zapata-Lamana

**Affiliations:** 1 Departamento de Educación y Humanidades, Universidad de Magallanes, Punta Arenas 62000000, Chile; javier.albornoz@umag.cl; 2 Department of Sports Sciences, Faculty of Sports Sciences, Universidad Europea de Madrid, 28670 Madrid, Spain; olga.barcelo@universidadeuropea.es; 3 Facultad de Ciencias de la Salud, Universidad Francisco de Vitoria, 28223 Madrid, Spain; sonia.garciamerino@ufv.es; 4 Department of Physiotherapy, Faculty of Sports Sciences, Universidad Europea de Madrid, 28670 Madrid, Spain; guillermo.garcia@universidadeuropea.es; 5 Escuela de Kinesiología, Facultad de Salud, Universidad Santo Tomás, Los Ángeles 4440000, Chile; icigarroa@santotomas.cl; 6 Escuela de Educación, Universidad de Concepción, Los Ángeles 4440000, Chile

**Keywords:** resistance training, exercise, educational centers, children, nutrition, healthy lifestyle, active rest

## Abstract

Background: Childhood obesity has tripled, reaching critical levels of malnutrition. This factor is directly associated with a poorer health-related quality of life of the child and adolescent population. This article presents the study protocol of the project “Strong schoolchildren with a healthy lifestyle” (EF-Salud), which seeks to analyze the effects of a multicomponent program based on muscle strength exercises, sleep nutritional recommendations, and the use of screens in Chilean educational centers with extremely cold weather. Methods: The study protocol of a randomized controlled trial with a pre- and post-test conducted according to the CONSORT statement is reported. The total sample (*n* = 144) will be schoolchildren from six different school years, four of which will perform an intervention and two control. Intervention group 1 (from two different school years) will receive a muscular strength exercise program in the classroom once a day from Monday to Friday for six months and nutritional, sleep, and use of screens recommendations once a week. Intervention group 2 (from two different school years) will receive a program of nutritional, sleep, and use of screens recommendations once per week for six months. The control group (from two different school years) will carry out their usual school day in relation to physical education classes. Before and after the intervention, the investigators will evaluate the cardiovascular risk, physical condition, and lifestyle related to sleep and use of screens. Expected results: The schoolchildren in intervention group 1 will obtain significant results in increased strength, decreased cardiovascular risk, improved sleep habits, and fewer hours of screen use compared to the other two groups.

## 1. Introduction

Global obesity has almost tripled from 1975 to date [1], reaching unacceptable levels of malnutrition. With more than 38.9 million children overweight and obese [2], it has become the most frequent modern pediatric chronic disease in the world [3]. These conditions have been associated for decades with the development of diseases, such as insulin resistance, type 2 diabetes mellitus, high blood pressure, fatty liver disease [4], and, recently, various types of cancer [5].

In Chile, total obesity (obesity + severe obesity) represents 31.0% of the school population and malnutrition due to excess (overweight + total obesity) 58.3%. The Magallanes Region presents one of the highest levels, with a total obesity prevalence of 33%, the schoolchildren in the fifth grade of primary education being those with the highest total obesity rate (36.3%) [6].

Childhood obesity is directly associated with a poorer health-related quality of life, resulting in greater school absenteeism [7]. In this sense, early detection and treatment of obesity in schoolchildren is the best approach to prevention [8,9]. The strategies that have been carried out for the treatment of obesity and childhood overweight such as modifications to the environment, changes in lifestyle habits [10], and interventions with the family and the educational center are considered effective against this disease [11]. These strategies consist of coordinating physical activities and giving nutritional and lifestyle recommendations within educational establishments [12,13].

Unfortunately, worldwide reports show that more than 80% of the child population does not reach the recommended levels of physical activity [14], and this percentage increases as schoolchildren age [15]. Additionally, the closure of schools and the lack of outdoor activities caused by the recent COVID-19 pandemic [16] affected schoolchildren in their lifestyle [17,18], altering sleep, screen time, diet, and especially physical activity [19]. For this reason, it should be possible to improve lifestyle through educational activities, such as increasing the amount of physical activity during the school day and promoting a healthy lifestyle based on a balanced diet [20,21,22], according to the physical activity recommendations recommended by experts [23].

Strategies such as active breaks correspond to short periods of physical activity of approximately 5 to 12 min carried out inside their classrooms [24]. These school programs promote physical well-being, thus improving physical activity levels, alertness, concentration in the classroom, and academic performance [25,26]. This methodology of the implementation of active breaks is mainly carried out by the teachers of the schoolchildren, using different types of educational strategies [27,28,29].

Over the past decade, there has been growing evidence of the “fat but fit” paradox, in which overweight and fit people have better health indicators and lower cardiovascular risk than healthy-weight, low-fit people [30]. In this regard, there is ample evidence of how physical fitness, and in particular muscle strength training, is being widely recommended by scientific organizations [31], as it is associated with a lower rate of cardiovascular risk factors and with numerous benefits for the physical condition and lifestyles of boys and girls [32,33,34], especially when accompanied by a good diet [35] and an ideal weight [36,37]. Some studies have shown that interventions in which only one intervention component is used seem to have smaller effects than multicomponent ones [38,39]. In fact, although there are many types of interventions in educational centers fighting against childhood obesity linked to malnutrition due to excess [40,41,42,43], inactive lifestyles [44,45], and low adherence to exercise [46,47], it is the multicomponent interventions that precisely combine resistance exercise, nutrition, and the adoption of healthy lifestyles by schoolchildren that show the best results in improving quality of life and physical condition and reducing the cardiovascular risk of children [13,44,48].

To date, according to the literature read, there is no study conducted in Chile that has analyzed the effects of a combined program of muscular strength exercises, eating habits, sleep, and the use of screens. Based on the evidence presented, the following research question arises: What effects provide a combined program of muscular strength exercises and nutritional, sleep, and screen use education on the physical condition, cardiovascular risk, and lifestyles of schoolchildren in the commune of Punta Arenas, Chile? In order to answer the question posed, the following objectives have been proposed:

*General objective*

To analyze the effects of a combined program of resistance exercises, nutritional recommendations, sleep, and use of screens in schoolchildren from educational establishments in Punta Arenas.

*Specific objectives (SO)*

**SO1:** To determine the physical condition, cardiovascular risk, and lifestyles at the beginning and at the end of the respective interventions in experimental group 1 (group that will carry out a combined program of muscular strength exercises and nutritional and lifestyle education related to sleep and use of screens), experimental group 2 (group that will carry out nutritional education and lifestyle activities related to sleep habits and use of screens), and the control group (group that will carry out the usual school day concerning physical education classes).

**SO2:** To compare the groups (intragroup differences) and between the groups (intergroup differences) in physical condition, cardiovascular risk, and lifestyles after both interventions.

**SO3:** To determine the benefits of the intervention of experimental group 1 in physical condition, cardiovascular risk, and lifestyles that allow making relevant and evidence-based decisions on the health of schoolchildren.

The following research hypotheses (Rh) are proposed:

**Rh1:**  *A combined program of muscular strength exercises and nutritional education improves the physical condition, cardiovascular risk, and lifestyles of schoolchildren from educational establishments in Punta Arenas.*

**Rh2:**  *A combined program of muscle strength exercises, nutritional and lifestyle education related to sleep habits, and the use of screens will present better results in improving physical condition, cardiovascular risk, and lifestyles than just education on nutrition and lifestyle related to sleep habits and use of screens or to only carry out the usual school day of physical education classes of schoolchildren in educational establishments in Punta Arenas.*

## 2. Materials and Methods

### 2.1. Experimental Design

**Figure 1:**
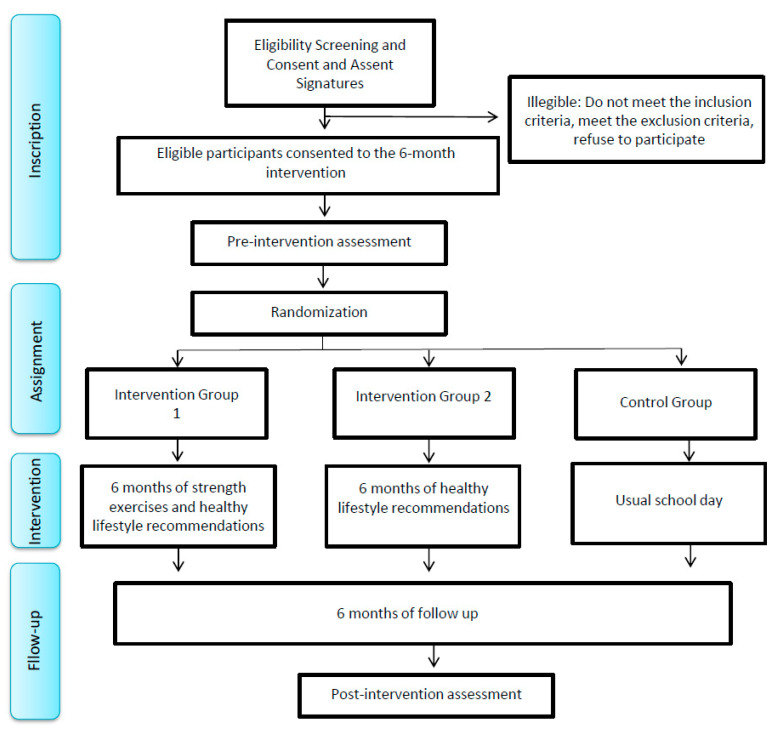
Flow diagram of study implementation.

This design (Figure 1) is a study protocol through a randomized controlled trial with a pre- and post-test, following the CONSORT statement [49].

The study is based on the Singapore and Helsinki declarations and all participants will be asked to voluntarily sign a consent form. The protocol was presented to the ethical-scientific committee for human beings of the University of Magallanes and approved with the code N^0^ 005/SH/2023.

### 2.2. Participants

The participants will be students of the 2nd formative cycle (fifth basic year) from two educational establishments in the commune of Punta Arenas, Magallanes Region, Chile. The ages selected for the research correspond to the highest percentage of schoolchildren with obesity and overweight in the region. The sample size will be 144 children (48 for intervention group 1, 48 for the intervention group 2, and 48 for the control group), which was obtained considering 50% heterogeneity, a margin of error of 5%, and a level of 95% confidence. The sample size was calculated a priori using G*Power 3.1.9.7. A total of 132 participants are required (22 in each course) considering α = 0.05, 1-β = 0.8, and an effect size of (ES) = 0.8. Considering the possibility of dropout, 12 more people were added (total *n* = 144; *n* = 24 in each course).

### 2.3. Inclusion and Exclusion Criteria

The inclusion and exclusion criteria will be schoolchildren with physical characteristics that allow them to carry out the evaluations (Table 1).

## 3. Procedure

Initial contact will be made with the director of education of the University of Magallanes for project approval and with the directors of the selected educational centers in order to inform them of the objectives and procedures of the intervention.

After having the authorization of the Ethics Committee of the University of Magallanes, the Department of Education, and the directors of the educational centers, training will be carried out for the research group to unify the data collection protocol and the intervention. Then, the teachers of the educational center will be trained, giving them all the information about the project, manuals, and guidelines for the activities to be carried out. They will receive the Gantt chart (Table 2).

At the end of the training, the schoolchildren will meet to give them a complete description of the project with its objectives and authorizations, and they will be invited to be part of it, for which they must sign an assent form. On the other hand, the researchers will attend a meeting with parents or legal guardians of the educational center in which they will receive all the background of the project, explaining all the steps to follow, objectives, risks, and expected achievements for the students. The researchers will invite them to give permission for the students to participate in the program by signing their consent.

### 3.1. Training for Project Collaborators

A preparation session will be held in which the project collaborators (kinesiologist, nutritionist, and psychologist) will be taught the data collection and test protocols. There will also be a tour of the facilities and they will be shown where the anthropometric tests (gym and room of the establishment) and the tests (computer room of the educational center) will be taken. In addition, the classrooms of fifth-grade students will be visited where strength exercises will be carried out and the classrooms where nutritional talks on sleep and the use of screens will be given.

### 3.2. Training for Teachers of the Educational Center

There will be a talk to the head teachers and the subject teachers that fifth-grade students have. Teachers will be instructed with the same information that will be given to schoolchildren, including nutritional recommendations, teaching the types of food intake and caloric expenditure, lifestyle, sleep habits, and screen time. In addition, they will be taught the exercise routine with elastic bands and with their body weight that schoolchildren will perform daily.

### 3.3. Information Collection

The data will be collected through self-report forms and physical examinations carried out by the project researchers in a space provided by the educational center conditioned with temperature, humidity, and privacy. The characterization of the sample considers age, sex, course, place of residence, educational level of the parents, number of medical licenses in the last year, % of non-attendance, and use of medicines prescribed by a doctor.

The evaluations will be carried out one week before the intervention in the three groups.

### 3.4. Randomization

An intentional sample will be carried out with randomization by course of the two selected establishments.

Each educational establishment has three fifth-grade courses each, with similar socio-educational characteristics for their level. The six courses will be randomized (24 subjects per course), of which two courses will represent intervention group 1, two courses will represent intervention group 2, and two courses will represent the control group.

### 3.5. Program: “Strong Schoolchildren with Healthy Lifestyle” (EF-Salud)

#### 3.5.1. Intervention Groups

##### Group 1: Strength Training + Nutritional, Sleep, and Screen Use Recommendations (ST + NSSR)

Co-investigators of the project will directly supervise the interventions, which will be carried out on the premises of the educational centers. The management of participants in the presence of adverse events will follow the emergency protocols established by the educational center.

The strength intervention protocol will consist of mobilization exercises and strength exercises of the upper and lower extremities using elastic bands. The students will carry out specific exercises to strengthen the muscles of the shoulders, arms and forearms, hips, thighs, and legs designed by the principal researcher (a physical education teacher who is an expert in training) together with a kinesiologist who is an expert in therapeutic exercise, who will be in charge of teaching schoolchildren and teachers to correctly perform the exercises once a day for 14 min from Monday to Friday.

The nutritional recommendations will be given by a nutritionist once a week, with a duration of 10 min, which will reveal the importance, types, frequency, and portions of healthy eating for the ages and characteristics of the schoolchildren.

The lifestyle recommendations will be given by a psychologist once a week for a period of 10 min, informing the schoolchildren about the lifestyle they should follow on a daily basis, screen time, sleep time, etc. (Table 3).

**Strength exercise program**

Bilateral and unilateral strength exercises [50,51] have been selected according to frequency, intensity, and volume [43], due to their potential health benefits [52] and their efficacy regarding the increase in lean mass, the decrease in cardiovascular risk factors, ease of application, and short duration [53,54,55,56].

*Warm-up*

The warm-up consists of joint mobility exercises for the wrists, elbow joint, shoulders, hip, knee, and ankle. It will last 4 min.

*Main exercises*

The participants will perform upper extremity exercises using an elastic resistance band [57]. The choice of this rubber will be made by the expert, where the resistance does not allow them to exceed 10–12 repetitions. There will be a familiarization session with Sci-Sport elastic bands of different sizes and resistances (XS, S, M, L, XL, XXL).

During the first two months, the XS band will be used, then during the third and fourth months the S-band will be used, and finally, during the fifth and sixth months, the M band will be used [57]. The exercises will have a moderate intensity [58] rating of 4 to 5 according to the Borg CR10 scale or modified Borg dyspnea scale [59] with a total duration of 2 min.

Exercises to perform with the upper extremities:

− Elbow flexion. Main active muscle: biceps. The participants will perform 2 series of 10 repetitions with each arm.
− Elbow extension. Main active muscle: triceps. The participants will perform 2 series of 10 repetitions with each arm.

*Exercise description:*

− Each child will perform the elbow flexion exercise in a sitting position. The elastic band (circular) will be located at one end on one foot which will hold it, while the other end of the band will be taken by hand. The exercise consists of flexing the elbow (which will be resting on the thigh) 10 times. Once the ten repetitions are finished, they will perform the same exercise with the other arm and repeat it in two series.
− The elbow extension exercise will be performed with the same elastic band that was used in the previous exercise. This will be held at one end by the back of the chair and the other end of the band will be held by both hands. The exercise will be performed with the hands behind the head, extending the elbow above it and pointing toward the ceiling of the room. Two series of ten repetitions will be performed.

Exercises to perform with the lower extremities:

These exercises will last two minutes in total.

− Flexion–extension of hip and knee (squat). Main active muscles: quadriceps and gluteus magnus. The participants will perform 2 series of 10 repetitions of sitting down and getting up from a chair.
− Knee flexion. Main active muscle: biceps femoris. The participants will perform 2 series of 10 repetitions of performing a knee flexion, standing up.

*Exercise description:*

− The children will perform squats sitting on a chair with their feet separated between 10 and 20 cm from each other. They will stand up without help to reach the bipedal position, completing 2 series of 10 repetitions, with a 30 s break between series.
− The knee flexion will be performed with a circular elastic band, standing up on the side of a chair. The children will join their extremities, aligning the knees, one against the other, and proceed to flex the knee, bringing the leg back until a 90-degree angle is achieved. This exercise will be repeated 10 times with each leg, for two series.

*Stretching*

At the end of the exercises, the students will perform stretching exercises for the muscles worked in the session. A physical education teacher and a kinesiologist will give the instructions for the exercise program in the classroom.

In each session, the exercises will be reviewed according to the children’s capacity and the execution technique. The participants will receive a notebook with all the strength exercises explained in detail. In addition, the notebook will have recommendations for a healthy lifestyle and nutrition.

**Nutritional recommendations**

A nutritionist will give nutritional recommendations on Mondays at 9:00 a.m. for six months through an expository class with the support of a projector. In these workshops, the schoolchildren will be taught about carbohydrates, proteins, lipids, junk food, and sugar intake. They will receive a diptych with the types of meals recommended for children aged between 10 and 12 years in addition to unhealthy foods, such as carbonated drinks, refined sugars, etc. This information will be shared with their parents and guardians.

**Recommendations for sleeping habits and screen use:**

The recommendations will be given by a psychologist (co-investigator of the project) once a week for 10 min, informing the schoolchildren about the lifestyle they must carry out daily, the time they should use screens, and the time they should sleep.

The talks will be focused mainly on the use of screens, dream habits, and interpersonal relationships of coexistence.

The program will last six months within the educational center and will be carried out by a multidisciplinary team [13]. This intervention should improve the physical condition and lifestyle of the schoolchildren, and therefore reduce cardiovascular risk as has been shown in the literature where overweight children with high muscle strength have fewer cardiometabolic risk factors than their overweight peers with low muscular strength [32,60,61].

##### Group 2: Nutritional, Sleep, and Use of Screens Recommendations (NSSR)

The nutritional, sleep, and use of screens recommendations will be taught together with experimental group 1, so the application conditions will be the same.

##### Group 3: Control Group (CG)

The CG will carry out a usual school day in relation to physical education classes.

The program will last six months (Table 3) and will be carried out within the educational centers by a multidisciplinary team [13]. It is expected that this improvement can reduce cardiovascular risk, as has been shown in the literature where overweight children with high muscle strength have fewer cardio-metabolic risk factors than their overweight peers with low muscle strength [32,43,60,61].

### 3.6. Variables

#### 3.6.1. Cardiovascular Risk

*Central obesity:* It will be analyzed using a specific index highly correlated with visceral adiposity, the waist-to-height ratio (WtHr). The waist circumference will be measured, taking as reference the equidistant point between the last non-floating rib and the iliac crest [62,63].

*Nutritional status*: The nutritional status will be assessed using the Body Mass Index (BMI). These measurements will be carried out according to the standardized procedures described by the International Society for Cinematographic Anthropometry (ISAK) (58) and according to the Habicht method.

#### 3.6.2. Physical Fitness

*Lower limbs muscle strength*: It will be assessed with the long jump (cm).

*Upper limbs muscle strength*: It will be assessed with the handgrip test, using a digital dynamometer (kg).

In both evaluations, the reference values recommended by the Alpha Fitness battery for the school population will be used, in which low strength and high strength are defined according to age and sex [64,65,66].

#### 3.6.3. Lifestyle

*Screen time:* To assess screen time, the investigators will use a self-reported survey consisting of three questions: “How many hours a day do you usually watch television?”; “How many hours a day do you usually play video games on a tablet, computer, or cell phone?”; and “How many hours a day do you usually use a tablet, computer, or cell phone for purposes other than gaming, for example, email, chats, social networks, the internet, or homework?” [67].

*Sleep hygiene:* The questionnaire “Sleep Self-Report (SSR)” in its Spanish version will be used [68].

All evaluations will be carried out before and immediately after the six months of intervention. The data set used and analyzed during the study will be available from the corresponding author upon reasonable request.

### 3.7. Statistical Analysis

All analyses will be performed using SPSS v.25 (SPSS, Inc., Chicago, IL, USA). Missing data will be handled by intention-to-treat analysis (multiple imputation method). The description will be made as measures of central tendency and dispersion (continuous variables) and as percentages (categorical variables). Using the Shapiro–Wilk test, the normality of the data will be verified, and a two-step rank transformation will be applied to the non-normal variables. Homoscedasticity will be analyzed using Levene’s test. A 2-way repeated measures analysis of variance (ANOVA) will be used to determine the effects of the interventions. The effects of the model are the group (GE1; GE2; GC), the times (pre-test; post-test), and their interaction over time (time × group). Bonferroni’s post hoc test will be applied to identify statistically significant comparisons. The effect size will be determined using Cohen’s d (<0.2 insignificant; ≥0.2 and ≤0.49 small; ≥0.5 and ≤0.79 moderate; ≥0.8 large), considering *p* < 0.05 and GraphPad Prism v8.

## 4. Expected Results

It is expected that the combined exercise program of muscle strength, lifestyles, and nutritional education (group 1) will present better results in terms of improving physical condition, cardiovascular risk, and healthy lifestyle compared to group 2, with only nutritional and lifestyle recommendations, and to the control group.

## 5. Discussion

The objective of this project is to show the efficacy of a multicomponent program of strength exercises + nutritional recommendations, sleep, and use of screens related to cardiovascular risk and strength in schoolchildren aged 10–12 years in educational centers of Chile with extremely cold weather.

It is expected that the group in which the strength interventions and lifestyle recommendations are applied will obtain significant results in terms of strength, decreased cardiovascular risk, improvements in sleep habits, and fewer hours of screen use compared to the group that will carry out only the nutritional and lifestyle recommendations and the control group that carries out their usual school day.

Regarding muscle strength and cardiovascular risk, overweight children who increase their muscle strength decrease cardiometabolic risk, unlike overweight children who do not increase their muscle strength [32,69,70,71]. Muscle strength and nutritional status can be assessed through the WtHr and BMI, which are predictors of cardiovascular risk in the prepubertal stage [62,72,73]. Strength is a significant indicator of health, which can be assessed by handgrip strength for the upper limbs and long jump for the lower limbs, the latter being the most relevant because schoolchildren develop this kind of ability at an earlier age [74]. On the other hand, an increase in muscle mass and strength encourages physical activity, reducing sedentary lifestyle and improving anthropometric indices [75,76], which leads to better physical self-perception, improvement in self-esteem, and increased levels of movement [77,78]. Along the same lines, a higher level of muscle strength boosts daily energy expenditure and lipid oxidation, resulting in better anthropometric measurements, better nutritional status [79,80], and a decrease in cardiovascular risk [80,81], a condition opposite to children with lower levels of muscle strength associated with worse cardiorespiratory conditions [82].

*Intervention characteristics:*

The intervention will be carried out for six months, allowing relevant results maintaining high adherence to be achieved and not suffering losses in the sample [83]. The adherence may also be related to the participation of teachers at the educational center and parents/guardians because the intervention includes healthy lifestyle education at home and at the school center [84]. Another characteristic of the project is the implementation of the intervention in the educational center, providing effectiveness and little sample loss, according to previous studies [8]. The combined intervention of strength + nutritional recommendations should include lifestyle changes [85] to ensure that long-term effects are maintained [86]. The beneficial effects of the interventions that combine components [3] have been documented in both the short term (≤6 months) and long term (≥12 months) [85,87], as reported in a systematic review where interventions lasting six months or less presented significant effects in 77% of the cases. According to the components addressed, the modalities that showed the highest effectiveness were physical activity, recreational activities (100%), a nutrition and diet program (100%), education (100%), and behavioral therapy (65%) [13].

*Limitations and strengths:*

This paper presents some weaknesses that need to be expressed. The first corresponds to the time for carrying out the intervention because the schedules of educational centers are rigid and structured and could be affected by the integration of an intervention model inside the classroom within the school day [26]. Another weakness could be the heterogeneous level of collaboration among teachers for using traditional class time.

However, the main strengths of the intervention correspond to carrying out the intervention within school hours [88], which can guarantee that all children and adolescents have the opportunity to participate, eliminating access barriers, such as distance and/or economic resources for transfers. Another strength of carrying out the intervention within the school day is the support of the teachers, who can play an active role in monitoring the intervention by encouraging the creation of healthy habits from an early age [28,29].

*Impact of the intervention:*

This type of intervention that combines strength exercises and nutritional and lifestyle recommendations can generate short-, medium-, and long-term health benefits for children and adolescents, such as improved physical condition, motor skills, and concentration; decreased anxiety; and the adoption of healthy eating and lifestyle habits [89,90]. This can have a positive impact on their academic performance, cognitive function, and general health status [91], including the prevention of chronic non-communicable diseases, such as obesity, type 2 diabetes, and cardiovascular diseases [92].

These types of interventions become relevant in the current context where children and adolescents increased their obesity rates as a result of an unhealthy lifestyle added to the decrease in physical activity, a consequence of the COVID-19 pandemic [93].

*Practical applications:*

This intervention within the school day can be incorporated into the school curriculum as other countries have done [94,95]. Strength exercises can be included daily at the beginning of the school day, and the recommendations of healthy lifestyle and nutrition habits within the curriculum of all classes [96]. This work plan can be carried out with the use of mobile applications, which offer personalized exercise routines with different types of levels and objectives according to their achievements, and can also be in a playful format to further attract children and adolescents [97,98]. In addition, this type of intervention can promote the creation of a healthy school environment [10], which consists of public policies that restrict the sale of unhealthy foods within educational establishments. Also, it can promote nutrition and daily physical activity habits within the establishment by doing sports, dances, and exercises and contacting places that deliver healthy food [99,100,101]. Finally, this intervention can coordinate with local institutions the realization of extracurricular activities that present sports and physical activity programs [102].

## Figures and Tables

**Table 1:** Inclusion and exclusion criteria.

Inclusion Criteria	Exclusion Criteria		
- Current registration at the time of the study.	- Physical, mental, or cognitive health conditions that prevent the proper completion of a program of exercise.		
- Both sexes, fifth grade (10–12 years old).			
- Residence in Punta Arenas.	- Acute or chronic decompensated pathology.		
- Parents’ or tutors’ legal consent. - Assent of schoolchildren.	- Severe vision or hearing loss.		
	- Presence of vertigo or epilepsy.		
	- Be participating in a physical exercise program regularly outside the educational center.		

**Table 2:** Gantt Chart Intervention, Year 2024.

	March	April	May	June	July	Aug.	Sept.	Oct./Nov.
Recruitment of support researchers	x							
Training of support professionals	x							
Coordination meeting with the director of the educational center	x							
Coordination meetings with teachers from the educational center	x							
Training for teachers of educational centers	x							
Delivery and collection of consents to parents and/or legal guardians	x	x						
Delivery and collection of informed consents of schoolchildren	x	x						
Preintervention measurements	x							
Group randomization	x							
Intervention within the educational centers		x	x	x	x	x	x	
Post intervention measurements								x
Data analysis								x
Delivery of reports to educational centers								x

Aug., August; Sept., September; Oct., October; Nov., November.

**Table 3:** Intervention group and control group.

Intervention Group 1	Intervention Group 2	Control Group 3
Strength exercises *	Usual routine habits	Usual routine habits
1 Warm-up		
2 Elbow flexion		
3 Elbow extension		
4 Hip and knee flexo-extension		
5 Knee flexion		
6 Stretching		
Nutritional recommendations **	Nutritional recommendations **	Usual routine habits
1 Water	1 Water	
2 Carbohydrates	2 Carbohydrates	
3 Proteins	3 Proteins	
4 Sugars	4 Sugars	
5 Lipids	5 Lipids	
Sleep and screen use recommendations ***	Sleep and screen use recommendations ***	Usual routine habits
1 Sleep habits	1 Sleep habits	
2 Screen time	2 Screen time	

* The strength exercises will be performed every day from Monday to Friday and each session will last 14 min. ** The nutritional recommendations will take place once a week and will last 10 min. *** The sleep and screen use recommendations will take place once a week and will last 10 min.

## Data Availability

Data are available upon request due to ethical and privacy restrictions.

## References

[1] World Health Organization Obesidad y Sobrepeso 2021. https://www.who.int/news-room/fact-sheets/detail/obesity-and-overweight.

[2] (2021). Independent Expert Group of Global Nutrition Report. Informe de la Nutrición Mundial 2021. Asociacion Civil de Centros de Estudios Sobre Nutrición Infantil. http://cesni-biblioteca.org/fuente/global-nutrition-Report/.

[3] Nemet D., Ben-Haim I., Pantanowits M., Eliakim A. (2012). Effects of a combined intervention for treating severely obese prepubertal children. J. Pediatr. Endocrinol. Metab..

[4] Daniels S.R., Pratt C.A., Hayman L.L. (2011). Reduction of risk for cardiovascular disease in children and adolescents. Circulation.

[5] Renehan A.G., Tyson M., Egger M., Heller R.F., Zwahlen M. (2008). Body-mass index and incidence of cancer: A systematic review and meta-analysis of prospective observational studies. Lancet.

[6] Lira M. (2021). Informe Mapa Nutricional 2021. Junta Nacional de Auxilio Escolar y Becas (JUNAEB) [Internet]. www.juaneb.cl.

[7] Kesztyüs D., Wirt T., Kobel S., Schreiber A., Kettner S., Dreyhaupt J., Kilian R., Steinacker J.M., The “Komm mit in das gesunde Boot-Grundschule”-Research Group (2013). Is central obesity associated with poorer health and health-related quality of life in primary school children? Cross-sectional results from the Baden-Württemberg Study. BMC Public Health.

[8] Verrotti A., Penta L., Zenzeri L., Agostinelli S., De Feo P. (2014). Childhood obesity: Prevention and strategies of intervention. A systematic review of school-based interventions in primary schools. J. Endocrinol. Investig..

[9] Reinehr T. (2013). Lifestyle intervention in childhood obesity: Changes and challenges. Nat. Rev. Endocrinol..

[10] Seidell J.C., Halberstadt J. (2015). The global burden of obesity and the challenges of prevention. Ann. Nutr. Metab..

[11] Adab P., Pallan M.J., Lancashire E.R., Hemming K., Frew E., Barrett T., Bhopal R., Cade J.E., Canaway A., Clarke J.L. (2018). Effectiveness of a childhood obesity prevention programme delivered through schools, targeting 6 and 7 year olds: Cluster randomised controlled trial (WAVES study). BMJ.

[12] Wabitsch M., Moss A., Kromeyer-Hauschild K. (2014). Unexpected plateauing of childhood obesity rates in developed countries. BMC Med..

[13] Albornoz-Guerrero J., García S., de Sevilla G.G.P., Cigarroa I., Zapata-Lamana R. (2021). Characteristics of Multicomponent Interventions to Treat Childhood Overweight and Obesity in Extremely Cold Climates: A Systematic Review of a Randomized Controlled Trial. Int. J. Environ. Res. Public Health.

[14] Guthold R., Stevens G.A., Riley L.M., Bull F.C. (2020). Global trends in insufficient physical activity among adolescents: A pooled analysis of 298 population-based surveys with 1·6 million participants. Lancet Child Adolesc. Health.

[15] Mitchell J.A. (2019). Physical Inactivity in Childhood from Preschool to Adolescence. ACSM’S Health Fit. J..

[16] Solomonova E., Picard-Deland C., Rapoport I.L., Pennestri M.-H., Saad M., Kendzerska T., Veissiere S.P.L., Godbout R., Edwards J.D., Quilty L. (2021). Stuck in a lockdown: Dreams, bad dreams, nightmares, and their relationship to stress, depression and anxiety during the COVID-19 pandemic. PLoS ONE.

[17] Samji H., Wu J., Ladak A., Vossen C., Stewart E., Dove N., Long D., Snell G. (2022). Review: Mental health impacts of the COVID-19 pandemic on children and youth—A systematic review. Child Adolesc. Ment. Health.

[18] Madigan S., Eirich R., Pador P., McArthur B.A., Neville R.D. (2022). Assessment of Changes in Child and Adolescent Screen Time During the COVID-19 Pandemic: A Systematic Review and Meta-analysis. JAMA Pediatr..

[19] Burkart S., Parker H., Weaver R.G., Beets M.W., Jones A., Adams E.L., Chaput J., Armstrong B. (2022). Impact of the COVID-19 pandemic on elementary schoolers’ physical activity, sleep, screen time and diet: A quasi-experimental interrupted time series study. Pediatr. Obes..

[20] Sturm D.J., Kelso A., Kobel S., Demetriou Y. (2021). Physical activity levels and sedentary time during school hours of 6th-grade girls in Germany. J. Public Health.

[21] Colella D., Monacis D., Limone P. (2020). Active Breaks and Motor Competencies Development in Primary School: A Systematic Review. Adv. Phys. Educ..

[22] Moscatelli F., De Maria A., Marinaccio L.A., Monda V., Messina A., Monacis D., Toto G., Limone P., Monda M., Messina G. (2023). Assessment of Lifestyle, Eating Habits and the Effect of Nutritional Education among Undergraduate Students in Southern Italy. Nutrients.

[23] Alonso-Martínez A.M., Ramírez-Vélez R., García-Alonso Y., Izquierdo M., García-Hermoso A. (2021). Physical Activity, Sedentary Behavior, Sleep and Self-Regulation in Spanish Preschoolers during the COVID-19 Lockdown. Int. J. Environ. Res. Public Health.

[24] Mahar M.T., Kenny R.K., Shields A.T., Scales D.P., Collins G. (2006). Energizers Classroom-Based Physical Activities 3–5: The Way Teachers Integrate Physical Activity with Academic Concepts. https://thescholarship.ecu.edu/handle/10342/5945.

[25] Neil-Sztramko S.E., Caldwell H., Dobbins M. (2021). School-based physical activity programs for promoting physical activity and fitness in children and adolescents aged 6 to 18. Cochrane Database Syst. Rev..

[26] Watson A., Timperio A., Brown H., Best K., Hesketh K.D. (2017). Effect of classroom-based physical activity interventions on academic and physical activity outcomes: A systematic review and meta-analysis. Int. J. Behav. Nutr. Phys. Act..

[27] Peláez-Flor V., Prieto-Ayuso A. (2021). “Aprendo Moviéndome”: Active breaks program for primary education. Sport TK.

[28] Masini A., Marini S., Leoni E., Lorusso G., Toselli S., Tessari A., Ceciliani A., Dallolio L. (2020). Active Breaks: A Pilot and Feasibility Study to Evaluate the Effectiveness of Physical Activity Levels in a School Based Intervention in an Italian Primary School. Int. J. Environ. Res. Public Health.

[29] Sánchez López M., García López L.M., Ruiz Hermosa A. (2020). Fichas de Descansos Activos para Educación Infantil y Primaria. Guía para el Profesorado.

[30] Ortega F.B., Lavie C.J., Blair S.N. (2016). Obesity and cardiovascular disease. Circ. Res..

[31] Peña G., Heredia J.R., Lloret C., Martín M., Da Silva-Grigoletto M.E. (2016). Iniciación al entrenamiento de fuerza en edades tempranas: Revisión. Rev. Andal. Med. Deporte.

[32] Smith J.J., Eather N., Morgan P.J., Plotnikoff R.C., Faigenbaum A.D., Lubans D.R. (2014). The health benefits of muscular fitness for children and adolescents: A systematic review and meta-analysis. Sports Med..

[33] Bermejo-Cantarero A., Álvarez-Bueno C., Martínez-Vizcaino V., Redondo-Tébar A., Pozuelo-Carrascosa D.P., Sánchez-López M. (2021). Relationship between both cardiorespiratory and muscular fitness and health-related quality of life in children and adolescents: A systematic review and meta-analysis of observational studies. Health Qual. Life Outcomes.

[34] Lavie C.J., Kachur S., Sui X. (2019). Impact of fitness and changes in fitness on lipids and survival. Prog. Cardiovasc. Dis..

[35] Yu C.C.W., Sung R.Y.T., Hau K.-T. (2008). The effect of diet and strength training on obese children’s physical self-concept. J. Sports Med. Phys. Fit..

[36] Carbone S., Kirkman D.L., Garten R.S., Rodriguez-Miguelez P., Artero E.G., Lee D.-C., Lavie C.J. (2020). Muscular Strength and Cardiovascular Disease: An updated state-of-the-art narrative review. J. Cardiopulm. Rehabil. Prev..

[37] Artero E.G., Lee D.-C., Lavie C.J., España-Romero V., Sui X., Church T.S., Blair S.N.P. (2012). Effects of muscular strength on cardiovascular risk factors and prognosis. J. Cardiopulm. Rehabil. Prev..

[38] Wang Y., Cai L., Wu Y., Wilson R.F., Weston C., Fawole O., Bleich S.N., Cheskin L.J., Showell N.N., Lau B.D. (2015). What childhood obesity prevention programmes work? A systematic review and meta-analysis. Obes. Rev..

[39] Marker A.M., Steele R.G., Noser A.E. (2018). Physical activity and health-related quality of life in children and adolescents: A systematic review and meta-analysis. Health Psychol..

[40] Bacopoulou F., Landis G., Rentoumis A., Tsitsika A., Efthymiou V. (2017). Mediterranean diet decreases adolescent waist circumference. Eur. J. Clin. Investig..

[41] Sadeghirad B., Duhaney T., Motaghipisheh S., Campbell N.R.C., Johnston B.C. (2016). Influence of unhealthy food and beverage marketing on children’s dietary intake and preference: A systematic review and meta-analysis of randomized trials. Obes. Rev..

[42] Boyland E.J., Nolan S., Kelly B., Tudur-Smith C., Jones A., Halford J.C., Robinson E. (2016). Advertising as a cue to consume: A systematic review and meta-analysis of the effects of acute exposure to unhealthy food and nonalcoholic beverage advertising on intake in children and adults. Am. J. Clin. Nutr..

[43] Wu C., Xu Y., Chen Z., Cao Y., Yu K., Huang C. (2021). The effect of intensity, frequency, duration and volume of physical activity in children and adolescents on skeletal muscle fitness: A systematic review and meta-analysis of randomized controlled trials. Int. J. Environ. Res. Public Health.

[44] Mead E., Brown T., Rees K., Azevedo L.B., Whittaker V., Jones D., Olajide J., Mainardi G.M., Corpeleijn E., O’Malley C. (2017). Diet, physical activity and behavioural interventions for the treatment of overweight or obese children from the age of 6 to 11 years. Cochrane Database Syst Rev..

[45] Luttikhuis H.O., Baur L., Jansen H., A Shrewsbury V., O’Malley C., Stolk R.P., Summerbell C.D. (2009). Interventions for treating obesity in children. Cochrane Database Syst. Rev..

[46] Li D., Chen P. (2021). The Effects of Different Exercise Modalities in the Treatment of Cardiometabolic Risk Factors in Obese Adolescents with Sedentary Behavior—A Systematic Review and Meta-Analysis of Randomized Controlled Trials. Children.

[47] Chen T., Lin J., Lin Y., Xu L., Lu D., Li F., Hou L., Yu C.C.W. (2021). Effects of aerobic exercise and resistance exercise on physical indexes and cardiovascular risk factors in obese and overweight school-age children: A systematic review and meta-analysis. PLoS ONE.

[48] Diao H., Wang H., Yang L., Li T. (2020). The impacts of multiple obesity-related interventions on quality of life in children and adolescents: A randomized controlled trial. Health Qual. Life Outcomes.

[49] Schulz K.F., Altman D.G., Moher D. (2010). CONSORT 2010 statement: Updated guidelines for reporting parallel group randomized trials. Ann. Intern. Med..

[50] Suchomel T.J., Nimphius S., Bellon C.R., Stone M.H. (2018). The Importance of Muscular Strength: Training Considerations. Sports Med..

[51] Andersen L.L., Vinstrup J., Jakobsen M.D., Sundstrup E. (2017). Validity and reliability of elastic resistance bands for measuring shoulder muscle strength. Scand. J. Med. Sci. Sports.

[52] Pochetti J., Ponczosznik D., Rojas P., Testa N. (2018). Strength training in children and adolescents: Benefits, risks and recommendations. Arch. Argent. Pediatr..

[53] González-Rosalén J., Medina-Mirapeix F., Cuerda-Del Pino A., Moreno-Segura N., Gacto-Sánchez M., Martín-San Agustín R. (2021). Analysis of Compliance with Time under Tension and Force during Strengthening Exercises with Elastic Bands. Diagnostics.

[54] Folkins E., Sahni S., Ryan J., Wooden S., Bushby G., Radzinski C. (2021). Concentric and Eccentric Force Changes with Elastic Band and Isotonic Heavy Resistance Training: A Randomized Controlled Trial. Int. J. Sports Phys. Ther..

[55] Ocampo N.V., Fredy Ramírez-Villada J., De Revisión A. (2018). El efecto de los programas de fuerza muscular sobre la capacidad funcional. Revisión sistemática Effects of muscular strength training programs on functional performance: Systematic review. Rev. Fac. Med..

[56] Wick K., Kriemler S., Granacher U. (2021). Effects of a Strength-Dominated Exercise Program on Physical Fitness and Cognitive Performance in Preschool Children. J. Strength Cond. Res..

[57] Sciences du Sport|Évaluation de la Résistance des Bandes Élastiques Sci-Sport Lors d’essais de Traction en Laboratoire. https://www.sci-sport.com/articles/evaluation-de-la-resistance-des-bandes-elastiques-sci-sport-lors-d-essais-de-tractions-en-laboratoire-060.php.

[58] División de Nutrición AF y O (2022). Medición de la Intensidad de la Actividad Física. Centers for Disease Control and Prevention. https://www.cdc.gov/physicalactivity/basics/measuring/index.html.

[59] Williams N. (2017). The Borg Rating of Perceived Exertion (RPE) scale. Occup. Med..

[60] Ramírez-Vélez R., Peña-Ibagon J.C., Martínez-Torres J., Tordecilla-Sanders A., Correa-Bautista J.E., Lobelo F., García-Hermoso A. (2017). Handgrip strength cutoff for cardiometabolic risk index among Colombian children and adolescents: The FUPRECOL Study. Sci. Rep..

[61] Albornoz-Guerrero J., Zapata-Lamana R., Reyes-Molina D., Cigarroa I., García Pérez de Sevilla G., García-Merino S. (2021). Overweight/Obese Schoolchildren with Low Muscle Strength Have a Lower Cardiorespiratory Capacity and Greater Cardiovascular Risk: Results of the School Health Survey of the Extreme South of Chile 2019. Children.

[62] Lo K., Wong M., Khalechelvam P., Tam W. (2016). Waist-to-height ratio, body mass index and waist circumference for screening paediatric cardio-metabolic risk factors: A meta-analysis. Obes. Rev..

[63] García J., Cárdenas A., Burgos S., Santiago C., Hernández F., Sanz V., Fernandez-Del-Valle M., Rubio M., Pérez M. (2019). Estilo de vida y distribución de grasa en adolescentes asmáticos y sanos. Rev. Int. Med. Cienc. Act. Física Deporte.

[64] Habicht J.-P. (1974). Estandarización de metodos epidemiológicos cuantitativos sobre el terreno. Boletín Oficina Sanit. Panam. (OSP).

[65] Aber L., Brown J.L., Jones S.M., Berg J., Torrente C. (2011). School-based strategies to prevent violence, trauma, and psychopathology: The challenges of going to scale. Dev. Psychopathol..

[66] Batería ALPHA-Fitness: Test de Campo para la Evaluación de la Condición Física Relacionada con la Salud en Niños y Adolescentes. https://scielo.isciii.es/scielo.php?script=sci_arttext&pid=S0212-16112011000600003.

[67] Aguilar M.M., Vergara F.A., Velásquez E.J.A., García-Hermoso A. (2015). Physical activity, screen time and sleep patterns in Chilean girls. An. Pediatría (Engl. Ed.).

[68] Orgilés M., Owens J., Espada J.P., Piqueras J.A., Carballo J.L. (2013). Spanish version of the Sleep Self-Report (SSR): Factorial structure and psychometric properties. Child Care Health Dev..

[69] Artero E.G., Ruiz J.R., Ortega F.B., España-Romero V., Vicente-Rodríguez G., Molnar D., Gottrand F., González-Gross M., Breidenassel C., Moreno L.A. (2011). Muscular and cardiorespiratory fitness are independently associated with metabolic risk in adolescents: The HELENA study. Pediatr. Diabetes.

[70] Steene-Johannessen J., Anderssen S.A., Kolle E., Andersen L.B. (2009). Low muscle fitness is associated with metabolic risk in youth. Med. Sci. Sports Exerc..

[71] Grøntved A., Ried-Larsen M., Møller N.C., Kristensen P.L., Froberg K., Brage S., Andersen L.B. (2015). Muscle strength in youth and cardiovascular risk in young adulthood (the European Youth Heart Study). Br. J. Sports Med..

[72] de Quadros T.M.B., Gordia A.P., Silva L.R. (2017). Anthropometry and clustered cardiometabolic risk factors in young people: A systematic review. Rev. Paul. Pediatr..

[73] de Quadros T.M.B., Gordia A.P., Andaki A.C.R., Mendes E.L., Mota J., Silva L.R. (2019). Utility of anthropometric indicators to screen for clustered cardiometabolic risk factors in children and adolescents. J. Pediatr. Endocrinol. Metab..

[74] Smith J.J., Eather N., Weaver R.G., Riley N., Beets M.W., Lubans D.R. (2019). Behavioral Correlates of Muscular Fitness in Children and Adolescents: A Systematic Review. Sports Med..

[75] García-Hermoso A., Correa-Bautista J.E., Olloquequi J., Ramírez-Vélez R. (2019). Health-related physical fitness and weight status in 13- to 15-year-old Latino adolescents. A pooled analysis. J. Pediatr..

[76] Ryan R.M., Williams G.C., Patrick H., Deci E.L. (2009). Self-Determination Theory and Physical Activity: The Dynamics of Motivation in Development and Wellness. Hell. J. Psychol..

[77] Fox K.R., Corbin C.B. (2016). The Physical Self-Perception Profile: Devlopment and Preliminary Validation. J. Sport Exerc. Psychol..

[78] Lubans D.R., Plotnikoff R.C., Lubans N.J. (2012). Review: A systematic review of the impact of physical activity programmes on social and emotional well-being in at-risk youth. Child Adolesc. Ment. Health.

[79] Moliner-Urdiales D., Ruiz J.R., Vicente-Rodriguez G., Ortega F.B., Rey-Lopez J.P., España-Romero V., Casajús J.A., Molnar D., Widhalm K., Dallongeville J. (2011). Associations of muscular and cardiorespiratory fitness with total and central body fat in adolescents: The HELENA study. Br. J. Sports Med..

[80] García-Hermoso A., Ramírez-Campillo R., Izquierdo M. (2019). Is Muscular Fitness Associated with Future Health Benefits in Children and Adolescents? A Systematic Review and Meta-Analysis of Longitudinal Studies. Sports Med..

[81] García-Hermoso A., Cavero-Redondo I., Ramírez-Vélez R., Ruiz J.R., Ortega F.B., Lee D.-C., Martínez-Vizcaíno V. (2018). Muscular Strength as a Predictor of All-Cause Mortality in an Apparently Healthy Population: A Systematic Review and Meta-Analysis of Data From Approximately 2 Million Men and Women. Arch. Phys. Med. Rehabil..

[82] Fraser B.J., Blizzard L., Cleland V., Schmidt M.D., Smith K.J., Gall S.L., Dwyer T., Venn A.J., Magnussen C.G. (2020). Factors associated with persistently high muscular power from childhood to adulthood. Med. Sci. Sports Exerc..

[83] Zeller M., Kirk S., Claytor R., Khoury P., Grieme J., Santangelo M., Daniels S. (2004). Predictors of attrition from a pediatric weight management program. J. Pediatr..

[84] Braet C., Van Winckel M., Van Leeuwen K. (1997). Follow-up results of different treatment programs for obese children. Acta Paediatr..

[85] Nemet D., Barkan S., Epstein Y., Friedland O., Kowen G., Eliakim A. (2005). Short- and long-term beneficial effects of a combined dietary-behavioral-physical activity intervention for the treatment of childhood obesity. Pediatrics.

[86] Epstein L.H., Roemmich J.N., Raynor H.A. (2001). Behavioral therapy in the treatment of pediatric obesity. Pediatr. Clin. N. Am..

[87] Juliana Kain B., Fernando Vio D., Barbara Leyton D., Ricardo Cerda R., Sonia Olivares C., Ricardo Uauy D., Cecilia Albala B. (2005). School-based health promotion intervention for primary schoolchildren from casablanca, chile. Rev. Chil. Nutr..

[88] Waters E., De Silva-Sanigorski A., Burford B.J., Brown T., Campbell K.J., Gao Y., Armstrong R., Prosser L., Summerbell C.D. (2011). Interventions for preventing obesity in children. Cochrane Database Syst. Rev..

[89] Yoong S.L., Lum M., Wolfenden L., Jackson J., Barnes C., Hall A.E., McCrabb S., Pearson N., Lane C., Jones J.Z. (2023). Healthy eating interventions delivered in early childhood education and care settings for improving the diet of children aged six months to six years. Cochrane Database Syst. Rev..

[90] Martin A., Booth J.N., Laird Y., Sproule J., Reilly J.J., Saunders D.H. (2018). Physical activity, diet and other behavioural interventions for improving cognition and school achievement in children and adolescents with obesity or overweight. Cochrane Database Syst. Rev..

[91] Wright C.M., Duquesnay P.J., Anzman-Frasca S., Chomitz V.R., Chui K., Economos C.D., Langevin E.G., Nelson M.E., Sacheck J.M. (2016). Study protocol: The Fueling Learning through Exercise (FLEX) study—A randomized controlled trial of the impact of school-based physical activity programs on children’s physical activity, cognitive function, and academic achievement. BMC Public Health.

[92] Storz M.A. (2020). The COVID-19 pandemic: An unprecedented tragedy in the battle against childhood obesity. Clin. Exp. Pediatr..

[93] Steenblock C., Hassanein M., Khan E.G., Yaman M., Kamel M., Barbir M., Lorke D.E., Everett D., Bejtullah S., Lohmann T. (2022). Obesity and COVID-19: What are the Consequences?. Horm. Metab. Res..

[94] Mavilidi M.F., Lubans D.R., Morgan P.J., Miller A., Eather N., Karayanidis F., Lonsdale C., Noetel M., Shaw K., Riley N. (2019). Integrating physical activity into the primary school curriculum: Rationale and study protocol for the “Thinking while Moving in English” cluster randomized controlled trial. BMC Public Health.

[95] Watson A., Timperio A., Brown H., Hesketh K.D. (2017). A primary school active break programme (ACTI-BREAK): Study protocol for a pilot cluster randomised controlled trial. Trials.

[96] Wright R.R., Nelson R., Garcia S., Butler A. (2020). Health Behavior Change in the Classroom: A Means to a Healthy End?. J. Prim. Prev..

[97] Lee M., Lee H., Kim Y., Kim J., Cho M., Jang J., Jang H. (2018). Mobile App-Based Health Promotion Programs: A Systematic Review of the Literature. Int. J. Environ. Res. Public Health.

[98] Milne-Ives M., LamMEng C., de Cock C., van Velthoven M.H., Ma E.M. (2020). Mobile Apps for Health Behavior Change in Physical Activity, Diet, Drug and Alcohol Use, and Mental Health: Systematic Review. JMIR mHealth uHealth.

[99] Congdon P. (2019). Obesity and Urban Environments. Int. J. Environ. Res. Public Health.

[100] Pouliou T., Elliott S.J. (2010). Individual and socio-environmental determinants of overweight and obesity in Urban Canada. Health Place.

[101] Verger P., Saliba B., Guagliardo V., Bouhnik A.D., Eichenbaum-Voline S. (2007). [Individual social characteristics, municipal environment and the prevalence of weight problems in early childhood: A multilevel analysis]. Rev. D’épidémiologie Santé Publique.

[102] Ley Chile-Decreto 290 17-SEP-1984 Ministerio de Educación Pública-Biblioteca del Congreso Nacional [Internet]. https://www.bcn.cl/leychile/navegar?idNorma=11989.

